# Effectiveness of a motivational interviewing intervention on weight loss, physical activity and cardiovascular disease risk factors: a randomised controlled trial with a 12-month post-intervention follow-up

**DOI:** 10.1186/1479-5868-10-40

**Published:** 2013-03-28

**Authors:** Sarah J Hardcastle, Adrian H Taylor, Martin P Bailey, Robert A Harley, Martin S Hagger

**Affiliations:** 1 School of Sport and Service Management, University of Brighton, Denton Road, Eastbourne, ES BN20 7SP, UK; 2 Sport, Exercise and Health Sciences, University of Exeter, St. Luke’s Campus, Exeter, Exeter EX1 2LU, UK; 3 Health Psychology and Behavioural Medicine Research Group, School of Psychology and Speech Pathology, Curtin University, GPO Box U1987, Perth WA6845, Australia

**Keywords:** Motivational interviewing, Health promotion, Obesity, Blood pressure, Cholesterol, Physical activity, Diet

## Abstract

**Background:**

Intensive diet and physical activity interventions have been found to reduce cardiovascular disease (CVD) risk, but are resource intensive. The American Heart Association recently recommended motivational interviewing (MI) as an effective approach for low-intensity interventions to promote health-related outcomes such as weight loss. However, there is limited research evaluating the long-term effectiveness of MI-based interventions on health-related outcomes associated with CVD risk. The current research evaluated the effectiveness of a six-month low-intensity MI intervention in a UK primary-care setting in maintaining reductions in CVD risk factors at12 months post-intervention.

**Methods:**

Primary-care patients were randomised to an intervention group that received standard exercise and nutrition information plus up to five face-to-face MI sessions, delivered by a physical activity specialist and registered dietician over a 6-month period, or to a minimal intervention comparison group that received the standard information only. Follow-up measures of behavioural (vigorous and moderate physical activity, walking, physical activity stage-of-change, fruit and vegetable intake, and dietary fat intake) and biomedical (weight, body mass index [BMI], blood pressure, cholesterol) outcomes were taken immediately post-intervention and at a 12-month follow-up occasion.

**Results:**

Intent-to-treat analyses revealed significant differences between groups for walking and cholesterol. Obese and hypercholesterolemic patients at baseline exhibited significant improvements in BMI and cholesterol respectively among those allocated to the intervention group compared to the comparison group. Post-intervention improvements in other health-related outcomes including blood pressure, weight, and BMI were not maintained.

**Conclusions:**

The present study suggests that a low-intensity MI counselling intervention is effective in bringing about long-term changes in some, but not all, health-related outcomes (walking, cholesterol levels) associated with CVD risk. The intervention was particularly effective for patients with elevated levels of CVD risk factors at baseline. Based on these findings future interventions should be conducted in a primary care setting and target patients with high risk of CVD. Future research should investigate how the long-term gains in health-related outcomes brought about by the MI-counselling intervention in the current study could be extended to a wider range of health outcomes.

## Introduction

Obesity is prevalent in the western world [1] and leads to reduced life expectancy due to increased risk to chronic illness such cardiovascular disease [2]. Even small reductions in weight result in clinically-meaningful reductions in important cardiovascular risk factors such as body mass index, hypercholesterolemia, and hypertension [3,4]. Since over 80% of the population visit their general practitioner annually [5] primary care has been identified is an important existing network through which effective weight loss interventions can be administered to tackle obesity. Behavioural interventions that target obesity through changes in diet and/or physical activity have been shown to be effective in producing clinically-significant reductions in weight (approximately 2 to 3 kg) at 4 to 7 years of follow-up [3,6-8]. The drawback of such interventions is that they tend to be intensive and require considerable financial and human resources to implement meaning that they are out-of-reach for most primary-care medical services to roll-out on a large scale [9]. Maintenance of behavioural changes associated with weight loss is also challenging [10], and often impressive short-term improvements are not translated into long-term behavioural maintenance [11].

The American Heart Association recently evaluated interventions that promote physical activity and dietary lifestyle change [12], and recommended motivational interviewing (MI) as an effective approach for initial weight loss up to 6-months [13,14]. Specifically, MI interventions have resulted in increased physical activity [15-17], reduced caloric intake [18] and decreased body mass index (BMI) [17,19] among patients following the intervention. A review of eight randomised controlled trials (RCTs) involving MI interventions for weight loss found that a median of 60 minutes of counselling reduced BMI by 0.72 kg.m^-2^ (equivalent to approximately 2 kg in body weight) compared to usual care [13]. Across a range of health behaviours, MI has been found to be an effective, relatively low intensity intervention, at least in the short-term [20-23]. However, most intervention studies have not included evaluations of maintenance. For example, most (n= 9) of the 11 studies included in a recent meta-analysis of MI interventions for weight loss [24] had a study duration of 6-months or less. In order to guide practice, further evidence is needed on the extent to which intervention outcomes are maintained following cessation of the intervention [25]. The current study contributes to a gap in the literature on the sustained effects of MI on weight loss, physical activity and cardiovascular disease (CVD) risk factors 12-months post intervention.

Motivational interviewing is not based on a particular theory and a lack of research on the ‘active ingredients’ [26,27] of MI interventions has made it difficult to draw firm conclusions regarding the processes by which MI facilitates behaviour change [28]. At the heart of MI is its ‘spirit’ which refers to the style of interaction between the practitioner and client [29]. According to MI, the style of interaction should be one of *collaboration*, *evocation*, and *autonomy*[30]. *Collaboration* refers to the practitioner as a ‘supportive partner’ rather than a ‘persuasive expert’ and collaborative component of MI stands in contrast to more prescriptive, expert-driven interventions that are commonly implemented in the dietary and physical activity domains. In synergy with collaboration, the *evocation* component of MI involves the practitioner drawing out the client’s personal motives for behaviour change. In this way, the role of the practitioner is to elicit rather than impart wisdom and knowledge, drawing on the perceptions and values of the client [29]. The final component that makes up the ‘spirit’ is the emphasis on *autonomy* and personal choice, where the responsibility, ability and decision to make behavioural changes are entirely under the client’s control. Together, these components form the essence of MI and are nicely summarised in the phrase by Miller and Rollnick [31] “You have what you need (for behaviour change), and together we’ll find it” (p.134).

MI has been linked to constructs from a number of social-psychological models of health behaviours [32,33]. Specifically, MI has been shown to provide three of the key components (i.e., structure, autonomy support, and relatedness) to support psychological needs for competence, autonomy and relatedness based on self-determination theory [34,35]. The satisfaction of these needs through MI is likely to foster increased autonomous motivation for behaviour change, and, as such, more likely lead to long-term adherence to health-promoting behaviours [36-38]. MI has also been linked closely with the enhancement of self-efficacy from social cognitive theory [39,40]. Setting personally-meaningful goals, providing feedback, and exploring current and imagined futures are all MI strategies that have been adopted to enhance self-efficacy. Finally, MI has also been linked to increasing motivational readiness to change from the transtheoretical model [41,42]. For example, Hardcastle et al. [41] found that increase in stage of change predicted physical activity change following MI. Given that several common strategies employed within MI are focused on building motivation for change (i.e., agenda setting, decisional balance, assessing importance, and eliciting change talk), we would expect motivation to increase. In summary, based on the theoretical underpinnings of MI and the strategies employed, there is real promise that MI interventions are likely to promote long-lasting, sustained behaviour change. This is because of its central emphasis on eliciting personal motives for change, working through ambivalence, building confidence and promoting more autonomous forms of motivation.

Despite the promising findings for the effectiveness of MI for promoting lifestyle change in the short term [13-19], evidence has been lacking for the long term effectiveness of MI on behavioural and biomedical outcomes for overweight or obese people. Only two previous studies have reported the effects of MI interventions for weight loss beyond 6-months [9,43]. However, neither of these studies was conducted in the primary care setting targeting those with CVD risk factors. For example, the study by Elliot et al. [43] recruited a healthy overweight, but not obese, sample comprising mainly young men that could be considered relatively lower risk compared to obese people. In addition, although follow-up measures were reported to have been taken at 12 months, those in the MI condition were contacted by phone six-and ten-months post-intervention and offered additional meetings meaning that the true follow-up period was only two months. As such, this study does not qualify as a maintenance study due to an insufficient gap between the end of the intervention and follow-up to examine sustainability sufficiently. In contrast, Smith-West [9] used MI in conjunction with an intensive intervention group based on a 42-session behavioural treatment programme. While combining the two treatments might be considered clinically worthwhile, the study did not shed light on the effectiveness of MI as a stand-alone treatment. The participants were also volunteers recruited via social marketing channels and are likely to have been more motivated than patients identified for recruitment through primary care based on their CVD risk. In the current study we aimed to redress this problem by only targeting patients in a primary care clinic identified as at having at least one CVD risk factor.

### The present study

In the current study we seek to address the gap in the literature on the sustained effects of an MI intervention on weight loss, physical activity, and CVD risk factors at a 12-month post-intervention follow-up occasion. The main aim of the study was to assess whether changes in weight, BMI, physical activity, and CVD risk factors within the intervention group were maintained one-year later. The second aim was to explore the effect of counselling session attendance (i.e., dose) on maintenance outcomes. The final aim was to examine the effects of motivational interviewing on outcomes for sub-groups presenting with specific CVD risk factors at baseline. Specifically, we expected the intervention to lead to significant long-term increases in physical activity and reductions in weight, BMI, blood pressure, and cholesterol. We also hypothesised that those attending more sessions would be more likely to maintain changes at 12-months post intervention. Further, we anticipated that groups comprising patients with specific risk factors, and receiving the MI intervention, would exhibit substantially larger changes in outcomes related to these risk factors compared to those who did not receive the intervention. The current study makes a unique contribution to the literature in that it is the first study to examine the maintenance of physiological and behavioural outcomes following an MI intervention in patients with CVD risk factors, by allowing a 12-month gap between end of intervention and follow-up.

## Method

### Participants and recruitment

Approval was obtained from the local NHS Research Ethics committee and Research Governance committee. Participants were drawn from a patient electronic database held at a local primary care health centre. In order to be included in the trial, participants needed to be aged 18–65 years and needed to exhibit at least one of the following CVD risk factors; excess weight (BMI of 28 or more, based on a value used in the recruiting GP practice), hypertension (SBP/DBP at least 150/90 mmHg), or hypercholesterolemia (at least 5.2 mmol.l^-1^). Assuming a medium effect size based on the results of previous interventions adopting MI [9,18,43] and with alpha set at 0.05, sample size calculations determined a need for approximately 120 participants in each group at follow-up in order to have a 97% of detecting a net change in BMI of 2 kg.m^-2^ (SD = 4); an 80% chance of detecting a change in SBP of 7.0 mmHg (SD = 19) and a 73% chance of detecting a change of 4.0 mmHg (SD = 12) in DBP. These outcome variables and change values were selected for the current study as they have been shown in previous research to reflect clinically-significant improvements in cardiovascular health and reduced CVD risk [3,44].

Our aim was to recruit a total of approximately 350 patients to allow for subject attrition at follow-up. A total of 1439 patients were contacted by mail with an invitation letter and information sheet telling them about the study. Three hundred and fifty eight (28%) accepted the invitation to enter the study by completing a form and returning it in a stamped addressed envelope. Recruitment bias was calculated by comparing data from patient electronic records from those who entered the trial and those invited but declined the invitation. Those entering the trial were significantly older (*M* age = 51.10 years, *SD* = 0.58 vs. *M* age = 48.40 years, *SD* = 0.36; *t*=4.06, *p*<.001, *d* = 0.43); with a higher BMI (*M* = 33.99 kg.m^-2^, SD = 0.33 vs. 31.70 kg.m^-2^ SD = 0.17; *t*=6.23, *p*<.001, *d* = 0.66); lower SBP (*M* = 130.70 mmHg, *SD* = 0.79 vs. *M* = 133.00 mmHg, *SD* = 0.49; *t* = 2.48, *p*<. 05, *d* = 0.26) and lower cholesterol (*M* = 5.40 mmol.l^-1^, *SD* = 0.06 vs. *M* = 5.61 mmol.l^-1^, *SD* = 0.05; *t* =2.88, *p* <.001, *d* = 0.30). Those patients who accepted the invitation were randomised into the MI intervention and minimal intervention arms of the study. A statistician, who had no contact with the participants, was asked to develop a randomisation protocol such that participants were allocated to the MI intervention and minimal intervention groups by a ratio of 7:5. The randomisation protocol was stratified by gender and age based on patient records. The patients within each stratum were divided into blocks of 12 and then randomly allocated to the MI intervention and minimal intervention groups using computer generated random numbers by the predetermined ratio. A 7:5 ratio was used as we expected a greater attrition rate in the MI intervention group. This stratified randomisation schedule was necessary to avoid groups that are unbalanced by age and gender.

**Figure 1:**
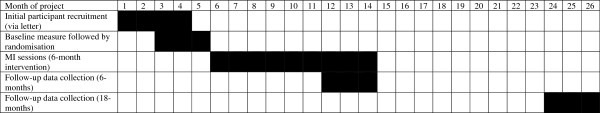
Timeline for the 26-month study.

Participants who wished to take part in the study were contacted by phone by a research assistant to ensure eligibility for inclusion in the trial and arrange a baseline assessment with a practice nurse at the health centre. The practice nurse was blind to the treatment allocated to each patient at baseline and subsequent assessments. After a baseline assessment conducted by trained nurses, all participants received a standard leaflet that provided information on exercise and nutrition. Participants randomly allocated to the MI intervention (treatment) were then given an appointment for their first face-to-face consultation with a physical activity specialist or registered dietician, with the opportunity to meet on up to four further occasions, for 20 to 30 mins, within the following 6-months. At 18 months post-baseline, all participants were invited by mail to attend a final assessment; this was again conducted by trained nurses who were blinded to the treatment allocation. The entire study lasted 26 months. This included the initial recruitment phase via an invitation letter to join the study. Once sufficient patients had consented to participate in the study, approximately two months after the initial letters had been sent, we began inviting patients for a baseline assessment. Once baseline assessments had been conducted, the MI counselling sessions began. The intervention lasted 6-months for each patient. Thereafter, we administered the 6- and 18-month follow-up assessments. A summary of the study timeline is given in Figure 1.

### MI counselling intervention

The counselling sessions were delivered by one trained physical activity specialist and one trained registered dietician. A patient-centred, tailored counselling intervention using was adopted incorporating principles and strategies from MI, integrated with a stage-matched approach [45]. Key strategies and techniques were used that adhere to the ‘spirit’ of MI [29]. Consistent with the underpinning ‘spirit’ of MI, personal motives to change (physical activity or diet) were identified by the patient and not imposed by the practitioner. The focus was on exploring ambivalence and eliciting self-directed ‘change talk’ [21]. Typical strategies adopted by the counselors to build motivation in those ambivalent about behavior change included agenda setting, exploration of the pros and cons, importance and confidence rulers. Strategies for those sufficiently motivated to change included strengthening commitment to change and negotiating a change plan [17].

### Minimal intervention group

Patients randomised to the minimal intervention group did not receive any MI counselling sessions. These participants were informed that they were part of a trial and received standard written information, in the form of a glossy A3 sized, double-sided poster that folded into an A5 leaflet on physical activity and diet produced by the Milton Keynes Primary Care Trust as a resource for health promotion. The leaflet includes lifestyle guidelines such as consuming five portions of fruit and vegetables per day, recommended fat intake and a recommendation to be physically active for 30 minutes, at least five times a week. The leaflet also lists the physiological and psychological benefits of increased physical activity. Finally, the leaflet included a food and physical activity quiz and advice depending upon scores. These participants also completed surveys containing self-reported measures of demographic, psychological, and behavioural variables. In addition, they were invited to the local primary-care surgery for biomedical measures to be taken at the requisite follow-up occasions. The protocol for the taking of the measures is detailed elsewhere [17].

### Counsellor training

The physical activity specialist and registered dietician participated in two four-hour training sessions conducted by the first author. The first session focused on the principles of MI including (1) that direct persuasion is ineffective in eliciting change (2) that it is the client’s task to articulate the reasons for change and resolve ambivalence, and (3) that the relationship between client and practitioner should be viewed as a partnership rather than as the traditional expert/recipient model [46]. The second session focused on strategies more suitable for those who are sufficiently motivated to change where the goal was to strengthen commitment to change and developing a plan to accomplish it. The practitioners went through a ‘menu’ of strategies they could use with patients depending on motivational readiness. These included agenda setting, exploring the pros and cons, exploring concerns/building confidence, providing information, asking key questions and negotiating a change plan. Following the training sessions, and during the first two weeks of consultations, the physical activity specialist and registered dietician each audio-taped three consultations. These formed the basis for a structured dialogue between the trainer and health professional, where the practitioners were able to discuss the difficulties of conducting MI and the trainer assisted with troubleshooting suggestions. Throughout the intervention period, monthly meetings took place to discuss issues about implementing MI and improving intervention fidelity. It is important to stress, however, that the MI practitioners were trained to a minimum acceptable standard prior to the commencement of the trial and all participants would have received the minimum requirements when it comes to content and delivery style in keeping with the MI intervention protocol. Any improvements in the professionals’ skills, during the course of the study would have been relatively minor, reflecting a ‘fine tuning’ of skills rather than wholesale changes.

### Outcome measures

Weight, height, systolic, and diastolic blood pressure (SBP/DBP), and fasting cholesterol, were assessed by a practice nurse as described previously. Self-reported physical activity was also assessed using the short interview version of the International Physical Activity Questionnaire (IPAQ) [47]. The IPAQ includes prompts for the intensity, frequency, and duration of respondents’ physical activity in the previous 7 days. A total physical activity score is calculated by adding up scores from the three intensity domains (vigorous, moderate and walking). Insufficient physical activity was defined as not meeting the recommendations as outlined in the Chief Medical Officer’s report [48], namely, less than 5 × 30 minutes of moderate-intensity physical activity per week. This equates to less than 600 MET-minutes per week for total physical activity. The IPAQ has acceptable reliability (Spearman’s rho = 0.8) and criterion validity (against the MTI accelerometer), which is comparable to most other self-report validation studies [49]. Physical Activity Stage of Change was also used as an outcome measure because readiness to change physical activity may increase even in the absence of PA behavioural change assessed by the IPAQ. Indeed, change in stage of change is consistent with a central purpose of MI, that is, to increase client readiness to change [29]. Physical Activity Stage of Change was assessed using a physical activity stage of change flowchart [50]. The flowchart enables the classification of participants in precontemplation, contemplation, preparation, action, or maintenance stages based on their dichotomous responses (‘yes’ or ‘no’) to five questions. Fat intake was assessed using a scale from the Dietary Instrument for Nutrition Education (DINE) [51]. The DINE is a food frequency questionnaire of 19 groups of food that account for around 70% of the fat and fibre in the typical UK diet according to the National Food Survey [52]. Each group of foods is assigned a score proportional to the fat or fibre content of a standard portion size [53]. The scores are weighted according to the frequency of consumption. The individual scores are added together to produce total scores for fat and fibre which can then be categorised into low (a score of 30 or less), medium or high intake (score greater than 40). In order to assess the validity of the DINE, a 4-day diet record, with a description of portion sizes was used as the reference method [47]. This reference method has been shown to be of acceptable validity relative to a 7-day weighed record [48].The five-a-day Community Evaluation Tool questionnaire (FACET) was used to determine fruit and vegetable consumption and has been shown to be correlated with consumption recorded via food diaries [54] and is considered sufficient for analysing group consumption patterns necessary for evaluating community interventions. A score of 5 indicates that patients were consuming five portions of fruit and vegetables a day. A more detailed description of all measures has been previously reported [17].

### Data analysis

Our data analyses were conducted in a series of steps – randomisation and attrition checks, main analyses testing the effects of the intervention on study outcomes, follow-up analyses examining dose effect, and, finally, intervention effects for subgroups of participants with specific individual risk factors.

We initially checked that patients were satisfactorily randomised to the MI intervention and minimal intervention groups by conducting two MANOVAs with the behavioural (total physical activity, walking, moderate physical activity, vigorous physical activity, stage of change, fat intake, fruit and vegetable intake), and biomedical (BMI, bodyweight, SBP, DBP, cholesterol, LDL, HDL, triglyceride) outcome variables as dependent variables and intervention group (MI intervention vs. minimal intervention) as an independent variables. In order to ensure that there was no bias in the samples arising from attrition we conducted two one-way MANOVAs with attendance (attended at 6-month follow up vs. failed to attend at 6-month follow-up) as the independent variable on the behavioural and biomedical outcome variables at baseline respectively.

For the main analysis, we used a series of 3 (time: baseline vs. 6-month follow-up vs. 18-month follow up) × 2 (group: MI intervention vs. minimal intervention) mixed-model ANCOVAs with repeated measures on the first factor and Bonferroni correction for multiple comparisons to assess the effects of the MI intervention on each of the behavioural and biomedical outcome variables separately. Age, gender, and smoking status were entered as covariates in each model.

In order to test for the effect of number of sessions attended on change in behavioural and outcome measures (baseline to 18-months), we used hierarchical linear multiple regression analyses. In the first step of the analyses, demographic variables (age, gender, smoking status) were entered as independent predictors. In the second step, the number of sessions, as a continuous independent variable (0–5)^[a]^ , was entered into the analysis. Analyses were conducted separately for each outcome and behavioural variable.

We also tested the effects of session attendance on outcomes using hierarchical multiple linear regression with each behavioural and biomedical outcome variables as a dependent variables with demographic (age, gender, and smoking status) and number of sessions as a continuous variable as independent variables entered in separate steps.

Finally, we aimed to examine the effects of the intervention on relevant biomedical outcome variables in groups of participants that exhibited corresponding specific risk factors at baseline (e.g., BMI and weight in participants classified as obese, DBP and SBP in participants classified as hypertensive etc.). We conducted a 3 (time: baseline vs. 6-month follow-up vs. 18-month follow up) × 2 (group: MI intervention vs. minimal intervention) mixed-model ANCOVA with repeated measures on the first factor and Bonferroni correction for multiple comparisons to assess the effects of the MI intervention on the outcome variables relevant to the specific elevated risk factor at baseline. The specific outcome (dependent) variables and accompanying high-risk groups were: BMI and weight in patients classified as obese (BMI ≥ 30 kg.m^-2^) at baseline; SBP in SBP hypertensive (>150 mmHg) patients, DBP in DBP hypertensive (>90 mmHg) patients, cholesterol in hypercholesterolemic patients (≥5.2 mmol.l^-1^), and overall physical activity in insufficiently active (<600 MET-mins.wk^-1^) patients. Age, gender, and smoking status were entered as covariates in each model.

We used a full intention-to-treat approach for the main analyses, the analyses of dose response, and the specific-risk factor subgroup analyses. For participants with missing data at follow-up, the last recorded value was used as the follow-up value (i.e., at baseline or 6 months). This is known as the last-observation-carried forward approach [55] and was particularly appropriate in the current study as the vast majority of participants (94.31%) dropped out between the baseline and 6-month follow-up occasion, rather than between the 6- and 18-month follow-up occasions, meaning that the analysis was unlikely to be affected by changed values that occurred participants who dropped out more recently.

## Results

### Baseline analyses and attrition checks

**Figure 2:**
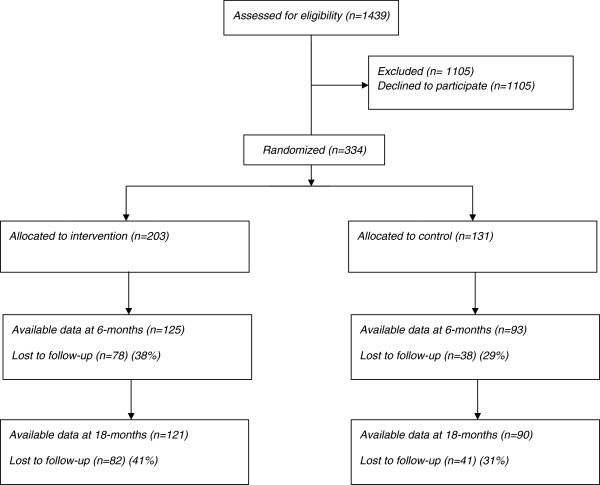
Flow of participants through trial.

A total of 334 patients completed the baseline assessment, of these 203 were randomised to the MI intervention and 131 to the minimal intervention groups. Table 1 provides an overview of the characteristics of the sample by condition at baseline. Figure 2 displays the flow of patients through the trial.

**Table 1:** Means and standard errors of baseline measures for total sample and by treatment group

**Variable**	**Total sample (n=334)**	**Intervention (n=203)**	**Control (n=131)**
Age (years)	50.22 (0.58)	50.10 (0.74)	50.41 (0.95)
Blood Pressure			
SBP (mmHg)	132.94 (0.98)	133.28 (1.25)	132.45 (1.57)
DBP (mmHg)	83.07 (0.57)	83.52 (0.72)	82.41 (0.91)
BMI (kg/m^2)^	33.65 (0.30)	33.67 (0.38)	34.28 (0.61)
Bodyweight (kg)	92.88 (0.93)	93.70 (1.20)	91.73 (1.50)
Cholesterol (mmol/L)	5.46 (0.06)	5.48 (0.08)	5.42 (0.09)
Triglycerides (mmol/L)	1.87 (0.07)	1.96 (0.09)	1.73 (0.09)
HDL (mmol/L)	1.49 (0.02)	1.46 (0.03)	1.53 (0.04)
LDL (mmol/L)	2.98 (0.07)	2.94 (0.09)	3.03 (0.10)
Fat intake (% per day)	23.80 (0.43)	23.85 (0.55)	23.72 (0.67)
Fruit and Vegetables (portions/ day)	6.49 (0.23)	6.41 (0.31)	6.88 (0.39)
Total PA (Met-min/week)^a^	1973.55 (133.83)	1828.45 (153.24)	2195.67 (243.83)
Vigorous PA (Met-min/week)^a^	634.14 (80.37)	585.76 (93.22)	709.27 (145.66)
Moderate PA (Met-min/week)^a^	483.17 (64.95)	437.05 (81.82)	554.39 (106.62)
Walking PA (Met-min/week)^a^	1087.92 (76.02)	1205.33 (137.36)	1011.92 (88.06)

*Note*. Values in parentheses are standard errors. ^a^ Met-min calculated by multiplying minutes of the respective activity in the past week by 8 (vigorous), 4 (moderate), and 3.3 (walking). Total PA (physical activity) is the sum of vigorous, moderate and walking Met-minutes.

MANOVAs assessing satisfactory randomisation to the intervention groups revealed no significant group differences for any of the outcome variables at baseline. It was notable that at baseline 99% of the participants were overweight or obese with 79% classified as obese, and 57% had elevated cholesterol (≥ 5.20 mmol.l^-1^). The mean number of counselling sessions attended over the 6-months was 2.00 (*SD =* 1.58), with 32%, 4%, 14%, 38%, and 12% attending 0,1,2,3 and 4 or 5 consultations respectively. The number of counselling sessions attended was rather variable and there are a number of reasons for this. Patients were not encouraged to attend a particular number of sessions (in keeping with the framework of MI) and resources did not allow for an ongoing check on counselling attendance. Given that the practitioners only worked two 4 hour blocks each week (and one block for each offered evening appointments), it’s likely that a number of patients could not attend the specific times available. In future studies, sufficient resources would be necessary to employ the practitioner for longer and more flexible hours in addition to exploring the reasons for non-engagement and low attendance. MANOVAs assessing the attribution bias in the sample revealed no significant overall differences for the behavioural (Wilks’ Lambda = 0.95, *F*(6,230) = 2.00, *p* = .067, η_p_^2^ = .050) and biomedical (Wilks’ Lambda = 0.99, *F*(8,252) = 0.44, *p* = .894, η_p_^2^ = .014) outcome variables. As the analysis for the behavioural outcome variables approached significance, we conducted univariate follow-up analyses to establish whether any significant differences were present. Those who attended the 6-month follow-up walked more at baseline (*M* = 1211 MET minutes, *SE*= 103) compared to those who dropped out (*M* = 863 MET minutes, *SE*= 101), *F*(1,235) = 4.50, *p* = .035, η_p_^2^ = .019. Attendees also reported higher stage of change levels (*M* = 3.40, *SE*= .11) compared to non-attendees (*M* = 3.01, *SE* = .15), *F*(1,235) = 4.26, *p* = .040, η_p_^2^ = .018. Finally, in terms of differences in demographic variables, those who attended were significantly older (*M* = 51.39, *SE* = 0.70) compared to those who dropped out at six months (*M* = 47.97, *SE* = 1.01), *t*(323) = 2.79, *p* = .006, *d* = 0.31. There was no difference in the proportion of males and females in the samples that attended and dropped out (χ^2^ = 0.04, *p* = .842)^[b]^ .

### Intervention effects

Results of the series of mixed-model ANCOVAs testing for the effects of the MI intervention on the behavioural and biomedical outcomes are shown in Table 2. For the behavioural dependent variables there were significant time × intervention group effects for walking, stage of change, and fat intake. For the biomedical outcomes, there were significant interaction effects for BMI, DBP, and cholesterol. We probed these interactions using univariate follow-up tests, again with Bonferoni adjustments for multiple comparisons, to locate the significant effects.

**Table 2:** Means and standard deviations for outcome measures by time and group based on an intent-to-treat analyses

**Outcome**	**Time**			**Analysis**^**a**^	
	**Baseline**	**6-Months**	**18-Months**	***Time x Group (F)***	**Effect size**^**b**^
Total Met Minutes/wk					
Intervention	1854.08 (2174.67)	2351.24 (2537.69)	3153.67 (3393.64)	2.59	.016
Control	2278.56 (2820.37)	2265.15 (2680.87)	3272.10 (3874.99)		
Walking Met Minutes/wk					
Intervention	996.07 (1116.59)	1195.54 (1277.60)	1265.14 (1352.25)	5.46**	.040
Control	1242.45 (1432.69)	1050.49 (1344.35)	1327.70 (1641.78)		
Moderate Met Minutes/wk					
Intervention	440.69 (1091.22)	531.72 (1150.98)	861.61 (1526.16)	1.18	.008
Control	576.15 (1159.23)	514.02 (968.80)	1086.24 (1670.45)		
Vigorous Met Minutes p/wk					
Intervention	590.05 (1294.38)	736.72 (1410.47)	1060.74 (2119.54)	1.01	.007
Control	746.55 (1672.04)	744.78 (1471.29)	972.04 (2023.38)		
Stage of Change					
Intervention	3.22 (1.36)	3.63 (1.35)	3.19 (1.61)	4.80**	.033
Control	3.47 (1.40)	3.47 (1.45)	2.87 (1.68)		
BMI					
Intervention	33. 66 (5.12)	33.53 (4.58)	33.68 (4.77)	4.11*	.028
Control	33.37 (4.47)	33.43 (5.07)	34.04 (4.88)		
Bodyweight					
Intervention	93.64 (15.93)	93.02 (15.55)	94.12 (15.66)	1.95	.013
Control	91.38 (16.88)	91.51 (17.41)	92.75 (17.37)		
SBP (mmHg)					
Intervention	133.12 (16.53)	130.25 (15.78)	128.98 (14.43)	1.71	.012
Control	132.41 (17.33)	131.81 (17.45)	129.96 (17.75)		
DBP (mmHg)					
Intervention	83.42 (9.63)	81.52 (8.57)	82.40 (9.03)	5.55**	.038
Control	81.92 (9.27)	82.70 (8.98)	82.81 (8.13)		
Cholesterol (mmol.l^-1^)					
Intervention	5.51 (1.01)	5.37 (1.05)	5.36 (1.03)	5.84**	.042
Control	5.39 (0.93)	5.37 (1.03)	5.52 (1.03)		
HDL (mmol.l^-1^)					
Intervention	1.46 (0.38)	1.41 (0.39)	1.33 (0.35)	0.02	.000
Control	1.52 (0.43)	1.47 (0.43)	1.39 (0.41)		
LDL (mmol.l^-1^)					
Intervention	2.96 (1.14)	3.06 (0.99)	3.28 (1.05)	1.29	.010
Control	3.01 (1.08)	3.27 (0.97)	3.48 (0.94)		
Triglycerides (mmol.l^-1^)					
Intervention	1.96 (0.79)	1.77 (1.25)	1.65 (1.01)	0.46	.004
Control	1.77 (1.02)	1.61 (0.79)	1.55 (0.78)		
Fat Intake (% fat intake per day)					
Intervention	23.87 (7.67)	22.93 (7.03)	22.97 (7.26)	4.41*	.028
Control	23.89 (7.70)	20.97 (6.46)	20.41 (5.96)		
Fruit & Vegetable Intake (portions per day)					
Intervention	6.31 (4.02)	7.33 (4.25)	6.30 (3.76)	0.78	.005
Control	6.94 (4.48)	7.58 (4.85)	6.23 (3.58)		

*Note*. Figures in parentheses are standard errors. ^a^*F*-ratio representing interaction effect of time by group on dependent variable; ^b^Partial eta-squared (η^2^_p_). *BMI* = Body mass index; *SBP* = Systolic blood pressure; *DBP* = Diastolic blood pressure; *HDL* = High density lipoproteins; LDL = Low density lipoproteins.

* *p* < .05.

** *p* < .01.

For the behavioural outcomes, there was a significant increase in walking between baseline and 6-months (*p* = .006, *d* = 0.24) and between baseline and 18-months (*p* = .032, *d* = 0.20) in the MI intervention group indicating sustained change for this variable over the follow-up period. Despite observed differences in the means, there were no significant univariate differences in walking for the minimal intervention group across time indicating that the intervention had no significant effect on walking scores for this group over time. For stage of change, there was a significant increase between baseline and 6-months (*p*<. 001, *d* = 0.33), which returned to near baseline levels at 18 months (*p* <. 001, *d* = 0.29) for the MI intervention group. For the minimal intervention group, there were no changes between baseline and 6-months and a significant decrease between baseline and 6-month (*p* = .016, *d* = 0.21) and 18-month (*p*< .001, *d* = 0.27) follow-up occasions indicating that stage of change actually decreased in the minimal intervention group but exhibited a significant, but short-lived, increase in the MI intervention group. Contrary to expectations, there was a significant decrease in dietary fat intake in between the baseline and 6-month follow-up period (*p*< .001, *d* = 0.43), a difference that was maintained at 18 months (*p*< .001, *d* = 0.38) for the minimal intervention group, whereas there was no difference in the MI intervention group.

For the biomedical outcomes, there was a significant increase in BMI between the baseline and 18-month (*p*= .001, *d* = 0.16) and between the 6- and 18-month (*p*= .007, *d* = 0.21) follow-up occasions in the minimal intervention group. There were no significant changes in BMI across the follow-up period for the MI intervention group. There was also a significant drop in DBP from baseline to 6-months (*p*<. 001, *d* = 0.29) in the MI intervention group, but DBP remained unchanged across the follow-up period for the minimal intervention group. There was a significant reduction in cholesterol between baseline and the 6-month (*p* = .008, *d* = 0.23) follow-up periods, a difference that was maintained at the 18-month follow-up occasion (*p* =. 015, *d* = 0.22) for the group. There was a significant increase in cholesterol between 6 and 18 months for the minimal intervention group (*p* = .007, *d* = 0.30).

### Dose response analysis

The hierarchical multiple regression analyses testing the effect of MI dose on the behavioural and biomedical outcome variables revealed no significant effect for MI dose, with the exception of triglycerides. The more sessions attended, the greater the reduction in triglycerides (β = −0.20, *t*= −2.54, *p*= .012, *d* = 0.28). There were significant effects for age on change in SBP (β = −0.20, *t*= 2.64, *p* = .009, *d* = 0.29) and reduction in HDL (β = −0.21,*t*= −2.77, *p*= .006, *d* = 0.30). This indicates that the younger the participant, the greater the improvement in HDL and the older the patient, the greater the improvement in SBP from baseline to 18-months. A significant effect was found for gender on HDL (β = 0.19, *t*=2.65, *p*= .009, *d* = 0.29) where males were more likely to gain greater improvements in HDL compared to females. Gender was also found to be a significant predictor of change in fat intake (β = − 0.15, *t*= −2.15, *p*= .033, *d* = −0.24) with females being more likely to consume more fat at 18-months. Finally, a significant effect was found for smoking status on HDL (β = 0.18, *t*= 2.34, *p*= .021, *d* = 0.26) whereby smokers had a greater improvement in HDL compared to non-smokers over the 18-months.

### Intervention effects by risk factor at baseline

Analyses of the effects of the intervention on specific relevant outcomes in subgroups of participants with elevated cardiovascular disease risk factors at baseline are presented in Table 3. For BMI in obese patients, there was a significant decrease in BMI between baseline and 6-months (*p* = .010, *d* = 0.26) but no differences between baseline and 18-months. In contrast there was a significant increase in BMI among patients in the minimal intervention group at the 18-month follow-up compared to both baseline (*p* = .015, *d* = 0.30) and 6-month (*p* = .037, *d* = 0.26) values. For cholesterol in hypercholesterolemic patients, here was a significant decrease in cholesterol levels between baseline and 6-months (*p* = .005, *d* = 0.31) and between baseline and 18-months (*p* = .003, *d* = 0.33) for participants in the MI intervention group indicating sustained change for this variable over the follow-up period. There were no significant changes in cholesterol levels for the minimal intervention group across time.

**Table 3:** Means and standard error of the mean for outcome measures by time and group based on CVD risk factor sub-group (intent-to-treat analyses)

**CHD risk factor**	**Time**			**Analysis**^**a**^	
	**Baseline**	**6-Months**	**18-Months**	***Time x Group (F)***	**Effect size**^**b**^
Obese					
Bodyweight				2.45	.022
Intervention (n= 133)	97.86 (1.16)	96.92 (1.17)	97.71 (1.21)		
Control (n= 93)	95.56 (1.39)	95.49 (1.40)	96.67 (1.45)		
Obese					
BMI≥30 kg.m^-2^				5.43*	.048
Intervention (n= 133)	35.37 (0.38)	35.00 (0.39)	35.04 (0.39)		
Control (n= 93)	34.68 (0.46)	34.75 (0.48)	35.22 (0.48)		
Hypertensive					
SBP>150 mmHg				1.04	.059
Intervention (n= 31)	150.49 (1.71)	141.82 (2.16)	138.23 (3.08)		
Control (n= 18)	153.71 (3.71)	152.19 (4.68)	144.74 (6.68)		
Hypertensive					
DBP>90 mmHg				2.10	.123
Intervention ( n= 46)	96.57 (0.97)	90.72 (1.33)	90.99 (1.30)		
Control (n= 24)	96.52 (1.58)	94.44 (2.15)	91.62 (2.11)		
Hypercholesterolemia					
(≥5.2 mmol.l^-1^)				3.01*	.036
Intervention (n= 107)	6.11 (0.07)	5.90 (0.08)	5.87 (0.08)		
Control (n= 67)	6.00 (0.08)	5.92 (0.11)	6.03 (0.11)		
Insufficiently					
Active					
<600 Met-mins .wk^-1^				0.15	.002
Intervention ( n= 78)	186.00 (22.50)	1124.95 (198.13)	2655.67 (372.16)		
Control (n= 44)	147.85 (30.59)	1053.88 (269.44)	2275.71 (506.09)		

*Note.* Figures in parentheses are standard errors. ^a^*F*-ratio representing interaction effect of time by group on dependent variable; ^b^Partial eta-squared (η^2^_p_). *BMI* = Body mass index; *SBP* = Systolic blood pressure; *DBP* = Diastolic blood pressure.

* *p* < .05.

## Discussion

Evidence has been lacking for the long term effectiveness of motivational interviewing (MI) on behavioural and biomedical outcomes for overweight or obese people. The current study contributes to a gap in the literature on the sustained effects of an MI intervention on weight loss, physical activity, and CVD risk factors among primary care patients at 12-months post-intervention. The main aim of the study was to assess whether changes in weight, BMI, physical activity, blood pressure, and cholesterol levels within the MI intervention group were maintained one-year later. The second aim was to explore the effect of counselling session attendance (i.e., dose) on maintenance outcomes. The final aim was to examine the effects of motivational interviewing on outcomes for sub-groups presenting with specific CVD risk factors at baseline. This is the first study to show that an MI intervention, delivered in the primary care setting, can contribute to a reduction in cholesterol and a significant increase in walking at both 6- and 12-months post-intervention, compared to an information-only group, for a sample with high levels of overweight or obesity. Only two previous studies [9,43] have reported the effects of MI interventions for weight loss beyond 6-months and both had substantial limitations curtailing their validity as long-term follow-up studies. The present study therefore offers important information on the potential effects that can be achieved by a targeted patient-centred lifestyle intervention delivered in a primary care setting.

In relation to the main aim to assess whether changes in weight, BMI, physical activity, and CVD risk factors within the MI intervention group were maintained at 12 months post-intervention, we found significant increases in walking in the MI intervention group that were maintained at 12 months. The MI intervention group also exhibited significant reductions in cholesterol during the intervention and this reduction was maintained 12-months later. In contrast, the minimal intervention group significantly increased cholesterol between the end of the intervention and 12-month follow-up. According to Ketola et al. [56], a clinically significant change in total cholesterol is classified as a decrease of 0.5 mmol/l. Therefore, the mean difference in our study of −0.16 mmol/l between groups at 18-months cannot be interpreted at clinically significant according to this index. However, the magnitude of the change found is comparable to changes reported in other studies that have examined the effectiveness of either individual lifestyle interventions [57,58] or of MI interventions on cholesterol [59,60]. There was also a significant increase in BMI from baseline to 18-months in the minimal intervention group but no significant change in BMI across follow-up for the MI intervention group. While the minimal intervention group increased BMI throughout the study, BMI was initially reduced in the MI group, but then reverted to baseline levels at 18 months.

In relation to the second aim, there were no significant effects for dose of MI on outcome measures from baseline to 18-months, with the exception of triglycerides, where the more sessions attended, the greater the improvement in triglycerides. Since any changes in the outcome variables were not associated with the number of sessions attended, these effects appear to be in response to a relatively low dose of MI. This indicates that a low-intensity MI can lead to significant improvements in cholesterol and physical activity up to one year following the intervention. Having a sample of overweight and mostly obese patients may have helped to give the study greater scope to detect such effects, compared with other studies that have mainly involved non-clinical populations, and this is one of the strengths of the present study.

In relation to the final aim involving sub-group analysis for those who were obese, hypertensive, hypercholesterolemic, and inactive at baseline, we found statistically significant differences in BMI at 6- and 12 months post-intervention for the MI intervention group, a trend that was not evident in the minimal intervention group. These findings are consistent with the effects of MI on weight reported in a recent meta-analysis of MI interventions targeting weight loss [24]. It should also be noted that the 18-month follow-up data were collected 12-months after cessation of the intervention in the current study. Given that the average attendance to the MI sessions was 2.00, the current study provides good evidence that a minimal intervention is effective in bringing about meaningful changes in some of the outcome variables. To speculate, the inclusion of relatively modest, low-cost ‘booster’ sessions may further bolster the effectiveness of the current intervention in the long term [61,62].

The ‘rebound’ effect, evident in the present study and commonly reported in behavioural intervention studies in which short-term changes in behaviour are lost at long-term follow-up [10,11], suggests that future research needs to concentrate on promoting the maintenance of behaviour change. There is evidence to suggest that maintenance of behavioural changes is more likely in trials that involved face-to-face contact than those that do not [63]. In addition, reviews on the maintenance of weight loss provide evidence to suggest that longer duration interventions (>6 months), that incorporate face-to-face contact are more effective in facilitating sustained weight loss [10,64]. The effects of MI could well be enhanced via the inclusion of booster sessions, beyond 6 months [61,62].

Also, reviews have revealed that both number and duration of MI sessions are related to behaviour change. For example, in studies that have used two MI sessions with 60 minutes per session, 81% of studies reported an effect [13,28]. The combined findings of the present study and Hardcastle et al. [17] showed a clear dose–response pattern up to 6-months, but these were all but lost by 12 months post-intervention. Further research is needed to explore the effectiveness of follow-up prompts (i.e., brief contact following the main intervention) on the maintenance of weight loss behavioural changes. Interventions that involve prolonged contact may be more effective than those based on high frequency contact [63]. The current study contributes to an insufficient body of literature on the maintenance of behavioural outcomes in physical activity and/or dietary intervention trials. Indeed, the recent review by Fjeldsoe [63] found that less than 20% of RCT’s published since 2000 have reported on behavioural maintenance and this was using a very conservative definition of maintenance (i.e., a follow-up period of 3 months or longer) [65]. Of these studies, only one that targeted both physical activity and diet included a 12-month follow-up, similar to the current study [66]; only one study recruited primary care patients [67] and almost half recruited healthy adults [68-70]. Furthermore, only two of the studies to meet criteria for inclusion in the review were conducted within the UK; both of which had relatively brief follow-ups of 3 and 6 months respectively [71,72].

### Limitations, avenues for future research, and conclusions

Despite the promising findings, the present study has some limitations. One limitation of the study concerns the low participation rate (28%). However, this was not entirely surprising given the opt-in procedure used and that among those at risk of CHD, many are not ready to change important lifestyle behaviours and therefore are unlikely to volunteer for such a trial. Nevertheless, although the patients who entered the trial might be more motivated for lifestyle change, they still needed to change and were recruited on the basis that they were at risk of CVD. As such we can be confident about the transferability of our findings to other patients at risk of CVD, albeit those that are more ready to change their lifestyle. Another limitation concerns the relatively low uptake of the intervention. Limited resources meant that we were unable to follow-up those participants with a reminder. However, this meant that the intervention had strong ecological validity as interventions carried out in practice are unlikely to have the resources or capacity to include extensive prompts or incentives to promote attendance. This limitation notwithstanding, we were still able to find significant effects of our intervention on the primary behavioural and biomedical dependent variables, making the present findings a relatively conservative estimate of the efficacy of such an intervention. A further limitation is that other important biomedical markers such as insulin and HbA1C were not measured. Such data would have been valuable to link the findings to similar literature on behavioural interventions involving pre-diabetic and diabetic populations. Similarly, measures of skinfold and other body composition outcomes would have provided further insights into how the MI intervention impacted on CVD risk. Such measures were considered, but were not undertaken, to avoid burdening the participant. The MI intervention was designed to be pragmatic and delivered in an ecologically-valid primary-care context. A second potentially limiting aspect of the study was the availability of resources to conduct a thorough process evaluation to determine: (a) fidelity of the intervention (including maintaining a patient-focus within the ‘spirit’ of MI, and coding of each session) and (b) which components of the intervention were most effective. During training of the intervention practitioners, sessions were tape recorded, the data coded and analysed, and feedback provided on the delivery of the MI intervention, but resource implications prevented a complete analysis. Future research should measure the most likely social psychological and motivational predictors of behaviour change hypothesized to mediate the effects of MI interventions [73,74]. This may include measures of self-efficacy [28,75,76], social support [77], autonomous forms of motivation from SDT [78], attitudes and perceived behavioural control from the Theory of Planned Behavior [79,80], and, motivational readiness from the Transtheoretical model [41,42] as important candidates to help explain the active ingredients of MI.

Nevertheless, a qualitative evaluation of this project [81] seeking to understand why some participants responded well to the intervention and maintained their reduced weight whilst others failed to change or failed to maintain change was undertaken. The qualitative evaluation pointed to differences in motivation and self-regulation. The lack of self-control identified by several participants was consistent with individual-difference theories of motivation which recognise that some individuals have lower capacity for sustained behaviour change and for resisting dominant responses. For example, recent work has demonstrated that people vary in their capacity to exercise self-control and this affects their ability to adhere to sustained and effortful behaviour change necessary to bring about weight loss through diet and physical activity [82-84]. Such individuals have low levels of self-control and are less likely to maintain behaviour change. However, such individuals are likely to benefit the most from MI interventions that are aimed at promoting autonomy, confidence, planning, and self-regulation. Indeed, research by Webber et al. [85] has shown that those with high levels of controlled motivation for weight loss at baseline lost significantly more weight in the MI group compared to those assigned to a standard behavioural weight loss intervention. Future work should take into account such individual differences and how to support those with lower levels of self-control. More research is needed to find the optimal number of consultations to promote autonomous forms of motivation and sustainable weight loss.

A further limitation of the study was the reliance on self-reported measures of physical activity and dietary behaviour [86]. The use of accelerometers and emerging dietary recording technologies could have provided a more objective understanding of behavioural changes associated with the intervention. Certainly, the significant reduction in fat intake self- reported by the minimal intervention group across time was unexpected and contrary to our expectations. One explanation for this difference may be that patients were central to what was discussed during counselling sessions and, perhaps feeling well informed (from general media attention) about fat intake, tended not to focus conversation on how to change this aspect of their lifestyle. Another explanation is that following the motivational interviewing session, participants in the intervention group became more realistic in their self-reporting of fat intake whereas those in the minimal intervention group may have underestimated their overall fat intake. Despite this unexpected finding, it is important to reinforce the clinically-relevant biomedical outcomes related to lipids found in the current study. Cholesterol was significantly improved for those in the intervention group compared to the minimal intervention group for those identified with hypercholesterolemia at baseline. In addition, for those identified as obese, there was a significant increase in BMI across time among patients in the minimal intervention group.

Finally, we did not set out to determine a full the cost-benefit analysis of the intervention. However, we recognise the importance of identifying the cost of such an intervention. The estimated cost of delivering the intervention was based on the hours of staff time (16 hours/week for 40 weeks) relative to the mean number of counselling sessions attended (n = 2) by all those randomised into the counselling group who attended the baseline assessment (n = 203). On average, each patient received one hour of counselling. Therefore, the average cost of delivering the intervention per patient was between £47 and £63 (depending on the expertise and experience of the practitioner. The cost is relatively cheap, given the potential health gains possible from using such an approach. Furthermore, given that motivational interviewing tends to involve around substantially less face-to-face contact time with clients compared to traditional programmes and appears to be equally effective, as evidenced by three meta-analytic reviews [87-89], the lower time commitment involved makes MI a potentially cost-effective intervention especially when resources are limited [90].

The current study contributes to a gap in the literature on the sustained effects of motivational interviewing on weight loss, physical activity and CVD risk factors a year following intervention. The MI intervention group sustained improvements in both walking and cholesterol. Also, for those with obesity and hypercholesterolemia at baseline, the MI intervention had a significant net favourable effect on BMI and cholesterol levels respectively.

## Conclusions

In conclusion, this is the first study to document the longer-term effects of adapted motivational interviewing, delivered in the primary care setting, on BMI, physical activity and related CVD risk factors. The MI intervention led to significant improvements in walking and cholesterol, which were maintained at 12-months. There was, however, no maintenance in other health-related outcomes including blood pressure, weight, and BMI. However, analyses of sub-groups of patients with elevated levels of specific risk factors showed evidence of maintained improvements over 12-months in the specific risk factor, although this was not the case for all sub-groups. Future research should seek to further examine the dose effects of number of MI sessions and also elucidate the mechanisms behind these changes, such as changes in variables associated with motivation and self-regulation such as autonomous motivation and self-efficacy. In addition, implementing MI interventions targeting participants with specific CVD risk factors and seeking to evoke changes in outcomes specific to the risk (e.g., reducing cholesterol in patients with hypercholesterolemia) may be a fruitful avenue for further research.

## Endnotes

^a^ As the number of intervention sessions was not normally distributed, we repeated the analysis using a logarithmic transformation of the intervention session variable to correct for violations of the assumption of normality. The size and pattern of effects were virtually identical to the untransformed analyses, so we have retained those figures.

^b^ For completion, we repeated the attrition analyses comparing baseline values for the demographic, behavioural, and biomedical outcome variables for those who were randomized to intervention groups at baseline and those who were retained at 18-month follow-up. Consistent with the MANOVA for the 6-month follow-up occasion, there were no significant overall differences in baseline values for the behavioural (Wilks’ Lambda = 0.97, *F*(6,229) = 1.22, *p* = .295, η_p_^2^ = .031) and biomedical (Wilks’ Lambda = 0.97, *F*(8,252) = 1.08, *p* = .378, η_p_^2^ = .033) variables between those who were retained at 18 months and those who dropped out. In terms of demographic variables, those who attended were significantly older (*M* = 52.19, *SE* = 0.70) compared to those who dropped out at 18 months (*M* = 47.29, *SE* = 1.01), *t*(322) = 4.22, *p*< .001, *d* = 0.47. There was no difference in the proportion of males and females in the samples that attended and dropped out (χ^2^ = 0.07, *p* = .809).

## Competing interest

There are no competing interests in relation to the study.

## Authors’ contributions

SJH conceived the study, secured the funding to conduct the research, developed the methods, conducted the data analysis, and took a lead role in drafting the manuscript. AHT gave advice on the data analysis and drafting the manuscript. MPB collated the data for analysis and assisted with drafting the manuscript. RAH assisted with securing the funding to conduct the research and participant recruitment and took a lead role in the project steering group. MSH conducted the data analysis and took a lead role in drafting the manuscript. All authors read and approved the final manuscript.

## References

[B1] Butland B Jebb S Kopelman P McPherson K, Thomas S, Mardell J, Parry V: Foresight: Tackling Obesities: Future choices- project report 2007 London: Government Office for Science

[B2] Jebb S Tackling the Weight of the Nation 2004 London: MRC Human Nutrition Research

[B3] Avenell A Broom J Brown TJ Poobalan A Aucott L Stearns SC Smith WCS Jung RT Campbell MK Grant AM Systematic review of the long-term effects and economic consequences of treatments for obesity and implications for health improvement Health Technol Assess 2004 8 21 10.3310/hta8210 15147610

[B4] Espeland M Pi-Sunyer X Blackburn G Brancati FL Bray GA Brigh R Reduction in weight and cardiovascular disease risk factors in individuals with type 2 diabetes-One year results of the Look AHEAD trial Diabetes Care 2007 30 1374 1383 17363746 10.2337/dc07-0048 PMC2665929

[B5] Walker A Maher J Coulthard M Goddard E Thomas M Living in Britain: Results from the 2000/01 General Household Survey 2001 London: Office for National Statistics

[B6] NICE Obesity guidance on the prevention, identification, assessment and management of overweight and obesity in adults and children 2006 London: National Institute for Health and Clinical Excellence

[B7] Shaw K Rourke P Del M Kenardy J Psychological interventions for overweight and obesity Coch Db Syst Rev 2005 2 CD003818 10.1002/14651858.CD003818.pub2 15846683

[B8] Knowler WC Barrett-Connor E Fowler SE Hamman RF Lachin JM Walker EA Reduction in the incidence of type 2 diabetes with lifestyle intervention or metformin New Eng J Med 2002 346 393 403 11832527 10.1056/NEJMoa012512 PMC1370926

[B9] Smith-West DS Dillo V Bursac Z Gore SA Greene PG Motivational interviewing improved weight loss in women with type 2 diabetes Diabetes Care 2007 30 1081 1087 10.2337/dc06-1966 17337504

[B10] Jeffery RW Drewnowski A Epstein LH Stunkard AJ Wilson GT Wing RR Hill DR Long-term maintenance of weight loss: current status Health Psychol 2000 19 5 16 10709944 10.1037/0278-6133.19.suppl1.5

[B11] Rothman AJ Toward a theory-based analysis of behavioural maintenance Health Psychol 2000 19 64 69 10709949 10.1037/0278-6133.19.suppl1.64

[B12] Artinian NT Fletcher GF Mozaffarian D Kris-Etherton P Van Horn L Lichenstein AH Kumanyika S Kraus WE Fleg JL Burke LE Interventions to Promote Physical Activity and Dietary Lifestyle Changes for Cardiovascular Risk Factor Reduction in Adults: A Scientific Statement from the American Heart Association Circulation 2010 122 406 441 10.1161/CIR.0b013e3181e8edf1 20625115 PMC6893884

[B13] Rubak S Sandbaek A Lauritzen T Christensen B Motivational interviewing: a systematic review and meta-analysis Br J Gen Prac 2005 55 305 312 PMC1463134 15826439

[B14] Britt E Hudson SM Blampied NM Motivational interviewing in health settings: a review Patient Educ Couns 2004 53 147 155 10.1016/S0738-3991(03)00141-1 15140454

[B15] Bennett JA Lyons KS Winters-Stone K Nail LM Scherer J Motivational interviewing to increase physical activity in long-term cancer survivors Nurs Res 2007 56 18 27 10.1097/00006199-200701000-00003 17179870

[B16] Carels RA Darby L Cacciapaglia HM Konrad Coit C Harper J Using motivational interviewing as a supplement to obesity treatment: A stepped-care approach Health Psychol 2007 26 369 374 17500624 10.1037/0278-6133.26.3.369

[B17] Hardcastle S Taylor AH Bailey M Castle R A randomised controlled trial on the effectiveness of a primary health care based counselling intervention on physical activity, diet and CHD risk factors Patient Educ Couns 2008 70 31 39 10.1016/j.pec.2007.09.014 17997263

[B18] Befort CA Nollen N Ellerbeck EF Sullivan DK Thomas JL Ahluwalia JS Motivational interviewing fails to improve outcomes of a behavioural weight-loss program for obese African American women: a pilot randomised trial J Behav Med 2008 31 367 377 10.1007/s10865-008-9161-8 18587639

[B19] Schwartz RP Hamre R Dietz WH Wasserman RC Slora EJ Myers EF Office-based motivational interviewing to prevent childhood obesity: A feasibility study Arch Pediat Adol Med 2007 161 495 501 10.1001/archpedi.161.5.495 17485627

[B20] Rollnick S Mason P Butler C Health behaviour change: a guide for practitioners 1999 Edinburgh: Churchill Livingstone

[B21] Aharonovich E Amrhein PC Bisaga A Nunes EV Hasin DS Cognition, commitment language, and behavioural change among cocaine-dependent patients Psychol Addict Behav 2008 22 557 562 19071981 10.1037/a0012971 PMC2605284

[B22] Lai DTC Cahill K Qin Y Tang J Motivational interviewing for smoking cessation Coch Db Syst Rev 2010 CD006936 10.1002/14651858.CD006936.pub2 20091612

[B23] Martins RK McNeil DW Review of Motivational Interviewing in promoting health behaviours Clin Psychol Rev 2009 29 283 293 10.1016/j.cpr.2009.02.001 19328605

[B24] Armstrong MJ Mottershead TA Ronksley PE Sigal RJ Campbell TS Hemmelgarn BR Motivational interviewing to improve weight loss in overweight and/or obese patients: a systematic review and meta-analysis of randomised controlled trials Obesity Rev 2011 12 709 723 10.1111/j.1467-789X.2011.00892.x 21692966

[B25] Eakin E Reeves M Winkler E Lawler S Owen N Maintenance of physical activity and dietary change following a telephone-delivered intervention Health Psychol 2010 29 566 573 20954778 10.1037/a0021359

[B26] Dombrowski SU Sniehotta FF Avenell A Johnston M MacLennan G Araújo-Soares A Identifying active ingredients in complex behavioural interventions for obese adults with additional risk factors: A systematic review Health Psychol Rev 2012 6 7 32 10.1080/17437199.2010.513298

[B27] Michie S Johnston M Theories and techniques of behaviour change: Developing a cumulative science of behaviour change Health Psychol Rev 2012 6 1 6 10.1080/17437199.2012.654964

[B28] Burke BL Arkowitz H Menchola M The efficacy of motivational interviewing: A meta-analysis of controlled clinical trials J Consult Clin Psychol 2003 71 843 861 14516234 10.1037/0022-006X.71.5.843

[B29] Miller WR Rollnick S Motivational interviewing: Preparing people for change 2002 2 New York: Guilford Press

[B30] Moyers TB Rollnick S A Motivational interviewing perspective on resistance in psychotherapy J Clin Psychol 2002 58 185 193 10.1002/jclp.1142 11793331

[B31] Miller WR Rollnick S Ten things that motivational interviewing is not Behav Cognit Psychother 2009 37 129 140 10.1017/S1352465809005128 19364414

[B32] Hagger MS Current issues and new directions in psychology and health: Physical activity research showcasing theory into practice Psychol Health 2010 25 1 5 20391203 10.1080/08870446.2010.502440

[B33] Hagger MS Self-regulation: An important construct in health psychology research and practice Health Psychol Rev 2010 4 57 65 10.1080/17437199.2010.503594

[B34] Deci EL Ryan RM The "What" and "Why" of goal pursuits: Human needs and the self-determination of behavior Psychol Inquiry 2000 11 227 268 10.1207/S15327965PLI1104_01

[B35] Markland D Ryan RM Tobin VJ Rollnick S Motivational interviewing and self-determination theory J Soc Clin Psychol 2005 24 811 831 10.1521/jscp.2005.24.6.811

[B36] Hagger MS Chatzisarantis NLD Causality orientations moderate the undermining effect of rewards on intrinsic motivation J Exp Soc Psychol 2011 47 485 489 10.1016/j.jesp.2010.10.010

[B37] Chatzisarantis NLD Hagger MS Biddle SJH Smith B Wang CKJ A meta-analysis of perceived locus of causality in exercise, sport, and physical education contexts J Sport Exerc Psychol 2003 25 284 306

[B38] Hagger MS Theoretical integration in health psychology: Unifying ideas and complimentary explanations Br J Health Psychol 2009 14 189 194 10.1348/135910708X397034 19236795

[B39] Bandura A Self-efficacy: Toward a unifying theory of behavioral change Psychol Rev 1977 84 191 215 847061 10.1037//0033-295x.84.2.191

[B40] Rohsenow DJ Monti PM Martin RA Colby SM Myers MG Motivational enhancement and coping skills training for cocaine abusers: Effects on substance abuse outcomes Addiction 2004 99 862 874 10.1111/j.1360-0443.2004.00743.x 15200582

[B41] Hardcastle S Blake N Hagger MS The effectiveness of a motivational interviewing primary-care based intervention on physical activity and predictors of change in a disadvantaged community J Behav Med 2012 35 318 333 10.1007/s10865-012-9417-1 22476812

[B42] Prochaska JO DiClemente CC Stages of change and process of change of self-change of smoking: Toward an integrative model J Consult Clin Psychol 1983 20 390 395 6863699 10.1037//0022-006x.51.3.390

[B43] Ellliot DL Goldberg L Keuhl KS Moe EL Breger RKR Pickering MA The PHLAME (Promoting Health Lifestyles: Alternative Models' Effects) firefighter study: Outcomes of two models of behavior change J Occup Environ Med 2007 49 204 213 10.1097/JOM.0b013e3180329a8d 17293760

[B44] Kelley GA Kelley KS Tran ZV Walking and resting blood pressure in adults: A meta-analysis Prev Med 2001 33 120 127 11493045 10.1006/pmed.2001.0860

[B45] Wilson PM Rodgers WM Fraser SN Murray TC Relationships between exercise regulations and motivational consequences in university students Res Q Exerc Sport 2004 75 81 91 10.1080/02701367.2004.10609136 15532364

[B46] Emmons KM Rollnick S Motivational interviewing in health care settings: Opportunities and limitations Am J Prev Med 2001 20 68 74 10.1016/S0749-3797(00)00254-3 11137778

[B47] Booth ML Assessment of physical activity: An international perspective Res Q Sport Exerc 2000 71 114 120 10.1080/02701367.2000.11082794 25680021

[B48] Department of Health At least five a week: Evidence on the impact of physical activity and its relationship to health. A report from the Chief Medical Officer 2004 London: Department of Health

[B49] Craig CL Marshall AL Sjostrom M Bauman AE Booth ML Ainsworth BE Pratt M Ekelund U Yngve A Sallis JF Oja P International physical activity questionnaire: 12-country reliability and validity Med Sci Sports Exerc 2003 35 1381 1395 10.1249/01.MSS.0000078924.61453.FB 12900694

[B50] Blair SN Dunn AL Marcus BH Carpenter RA Jaret P Active living everyday: 20 weeks to lifelong vitality 2001 Champaign, Illinois: Human Kinetics

[B51] Roe L Strong C Nei A Mant D Dietary intervention in primary care: validity of the DINE method for diet assessment Fam Pr 1994 11 375 381 10.1093/fampra/11.4.375 7895964

[B52] Department for the Environment Food and Rural Affairs (DEFRA) National food survey 2000: Annual report on food expenditure, consumption and nutrient intakes 2001 London: HMSO

[B53] Crawley H Food portion sizes (MAFF Handbook) 1994 2 London: HMSO

[B54] Ashfield-Watt PAL Welch AA Godward S Bingham SA Effect of a pilot community intervention on fruit and vegetable intakes: Use of FACET Pub Health Nutr 2007 10 671 680 17381948 10.1017/S1368980007382517

[B55] Shao J Zhong B Last observation carry-forward and last observation analysis Statistics in Medicine 2003 22 2429 2441 10.1002/sim.1519 12872300

[B56] Ketola E Sipila R Makela M Effectiveness of individual lifestyle intervention in reducing cardiovascular disease and risk factors 2000 32 239 251 10.3109/07853890009011767 10852140

[B57] Knutsen SF Knutsen R The Tromso survey: The family intervention study- the effectiveness of intervention on some coronary risk factors and dietary habits, a 6 year follow-up 1991 20 197 212 10.1016/0091-7435(91)90020-5 2057468

[B58] Oxcheck Study Group Effectiveness of health checks conducted by nurses in primary care: Results of Oxcheck study after one year 1994 308 308 312 PMC2539253 8124120

[B59] Ockene IS Herbert JR Ockene K Effect of physician-delivered nutrition counselling training and an office support program on saturated fat intake, weight and serum lipid measurements in a hyperlipidemic population 1999 159 725 731 10.1001/archinte.159.7.725 10218753

[B60] Mhurchu CN Margetts BM Speller V Randomised controlled trial comparing the effectiveness of two dietary interventions for patients with hyperlipidaemia Clinical Science 1998 95 479 487 10.1042/CS19980118 9748424

[B61] Chapman J Armitage CJ Evidence that boosters augment the long-term impact of implementation intentions on fruit and vegetable intake Psychol Health 2010 25 365 381 10.1080/08870440802642148 20204966

[B62] Luszczynska A Haynes C Changing nutrition, physical activity and body weight among student nurses and midwives: Effects of a planning intervention and self-efficacy beliefs J Health Psychol 2009 14 1075 1084 10.1177/1359105309342290 19858328

[B63] Fjeldsoe B Neuhaus M Winkler E Eakin E Systematic review of maintenance of behaviour change following physical activity and dietary interventions Health Psychol 2011 30 99 109 21299298 10.1037/a0021974

[B64] Lombard CB Deeks AA Teede HJ A systematic review of interventions aimed at the prevention of weight gain in adults Pub Health Nutr 2009 12 2236 2246 10.1017/S1368980009990577 19650959

[B65] Trials of Hypertension Prevention Collaborative Research Group The effect of a nonpharmacolgic intervention on blood pressure of persons with high normal levels. Results of the Trials of Hypertension Prevention, phase1 JAMA 1992 267 1213 1220 10.1001/jama.1992.03480090061028 1586398

[B66] Greaney ML Riebe D Garber CE Rossi JS Lees FD Burbank PA Clark PG Long-term effects of a stage-based intervention for changing exercise intentions and behaviour in older adults Gerontologist 2008 48 358 367 10.1093/geront/48.3.358 18591361

[B67] Prochaska JO Velicer WF Redding C Rossi J Goldstein M DePue J Plummer BA Stage-based expert systems to guide a population of primary care patients to quit smoking, eat healthier, prevent skin cancer, and receive regular mammograms Prev Med 2005 41 406 416 10.1016/j.ypmed.2004.09.050 15896835

[B68] De Vet E Oenema A Sheeran P Brug J Should implementation intentions interventions be implemented in obesity prevention: The impact of if-then plans on daily physical activity in Dutch adults Int J Behav Nutr Phys Act 2009 6 11 10.1186/1479-5868-6-11 19267889 PMC2662780

[B69] Jimmy G Martin BW Implementation and effectiveness of a primary care based physical activity counselling scheme Patient Educ Couns 2005 56 323 331 10.1016/j.pec.2004.03.006 15721975

[B70] Fries E Edinboro P McClish D Manion L Bowen D Beresford SA Ripley J Randomised trial of a low intensity dietary intervention in rural residents Am J Prev Med 2005 28 162 168 10.1016/j.amepre.2004.10.017 15710271

[B71] Clark M Hampson SE Avery L Simpson R Effects of a tailored lifestyle self-management intervention in patients with Type 2 diabetes Br J Health Psychol 2004 9 365 379 10.1348/1359107041557066 15296683

[B72] Lindsay S Smith S Bellaby P Baker R The health impact of an online heart disease support group: A comparison of moderated versus unmoderated support Health Educ Res 2009 24 646 654 10.1093/her/cyp001 19251770

[B73] Amireault S Godin G Vohl M Perusse L Moderators of the intention behaviour and perceived behavioural control-behaviour relationships for leisure-time physical activity Int J Behav Nutr Phys Act 2008 5 7 10.1186/1479-5868-5-7 18241339 PMC2275296

[B74] Lorentzen C Ommundsen Y Jenum AK Holma I The "Romsas in Motion" community intervention: Programme exposure and psychosocial mediated relationships to change in stages of change in physical activity Int J Behav Nutr Phys Act 2007 4 15 10.1186/1479-5868-4-15 17466077 PMC1871604

[B75] Lewis BA Forsyth LH Pinto BM Bock BC Roberts M Marcus BH Psychosocial mediators of physical activity in a randomized controlled intervention trial J Sport Exerc Psychol 2006 28 193 204

[B76] Hagger MS Biddle SJH Chow EW Stambulova N Kavussanu M Physical self-perceptions in adolescence: Generalizability of a hierarchical multidimensional model across three cultures Journal of cross-cultural Psychology 2003 34 611 628 10.1177/0022022103255437

[B77] Parks SE Housemann RA Brownson RC Differential correlates of physical activity in urban and rural adults of various socioeconomic backgrounds in the United States J Epidemiol Community Health 2003 57 29 35 10.1136/jech.57.1.29 12490645 PMC1732269

[B78] Chatzisarantis NLD Hagger MS Effects of an intervention based on self-determination theory on self-reported leisure-time physical activity participation Psychol Health 2009 24 29 48 10.1080/08870440701809533 20186638

[B79] Courneya KS Bobick TM Integrating the theory of planned behavior with the processes and stages of change in the exercise domain Psychol Sport Exerc 2000 1 41 56 10.1016/S1469-0292(00)00006-6

[B80] Hagger MS Anderson M Kyriakaki M Darkings S Aspects of identity and their influence on intentional behaviour: Comparing effects for three health behaviour Personality and Individual Differences 2007 42 355 367 10.1016/j.paid.2006.07.017

[B81] Hardcastle S Hagger MS “You can’t do it on your own”: Experiences of a motivational interviewing intervention on physical activity and dietary behaviour Psychol Sport Exerc 2011 12 314 323 10.1016/j.psychsport.2011.01.001

[B82] Baumeister RF Gailliot MT DeWall CN Oaten M Self-regulation and personality: How interventions increase regulatory success, and how depletion moderates the effects of traits on behavior J Pers 2006 74 1773 1801 10.1111/j.1467-6494.2006.00428.x 17083666

[B83] Fishbach A Friedman RS Kruglanski AW Leading us not unto temptation: Momentary allurements elicit overriding goal activation J Pers Soc Psychol 2003 84 296 309 12585805

[B84] Hagger MS Wood C Stiff C Chatzisarantis NLD The strength model of self-regulation failure and health-related behavior Health Psychol Rev 2009 3 208 238 10.1080/17437190903414387

[B85] Webber KH Gabriele JM Tate DF Dignan MB The effect of a motivational intervention on weight loss is moderated by level of baseline controlled motivation Int J Behav Nutr Phys Act 2010 7 4 10.1186/1479-5868-7-4 20157441 PMC2821313

[B86] Hagger MS Chatzisarantis NLD Assumptions in research in sport and exercise psychology Psychol Sport Exerc 2009 10 511 519 10.1016/j.psychsport.2009.01.004

[B87] Lundahl BW Kunz C Brownell C Tollefson D Burke BL A meta-analysis of motivational interviewing: Twenty-five years of empirical studies Res Soc Work Pract 2010 20 137 160 10.1177/1049731509347850

[B88] Hettema J Steel J Miller W Motivational interviewing Ann Rev Clin Psychol 2005 1 91 111 10.1146/annurev.clinpsy.1.102803.143833 17716083

[B89] Fjeldsoe BS Miller YD Marshall AL MobileMums: A Randomized Controlled Trial of an SMS-Based Physical Activity Intervention Ann Behav Med 2010 39 101 111 10.1007/s12160-010-9170-z 20174902

[B90] Lundahl BW Burke BL The effectiveness and applicability of motivational interviewing: A practice-friendly review of four meta-analyses J Clin Psychol 2009 65 1232 1245 10.1002/jclp.20638 19739205

